# Low‐energy diets before metabolic bariatric surgery: A systematic review of the effect on total body weight, liver volume, glycemia and side effects

**DOI:** 10.1111/obr.13876

**Published:** 2024-12-03

**Authors:** Inger Nilsen, Agneta Andersson, Anna Laurenius, Johanna Österberg

**Affiliations:** ^1^ Department of Food Studies, Nutrition and Dietetics Uppsala University Uppsala Sweden; ^2^ Center for Clinical Research Dalarna Falun Sweden; ^3^ Department of Dietetics and Speech Therapy, Mora Hospital, Mora Sweden; ^4^ Department of Surgery, Institute of Clinical Science, Sahlgrenska Academy University of Gothenburg Gothenburg Sweden; ^5^ Department of Gastroenterology and Hepatology, Unit of Clinical Nutrition and the Regional Obesity Center Sahlgrenska University Hospital Gothenburg Sweden; ^6^ Department of Surgery, Mora Hospital, Mora Sweden; ^7^ Department of Clinical Science and Education, Södersjukhuset Karolinska Institute Stockholm Sweden

**Keywords:** glucose concentration, insulin resistance, liver volume, low‐energy diet, metabolic bariatric surgery, preoperative, total body weight

## Abstract

There is no consensus regarding energy content or duration of hypocaloric diets used for preoperative optimization of patients before metabolic bariatric surgery. In this systematic review, we aimed to compare the effect of different hypocaloric diets on reductions in total body weight, liver volume, glucose and insulin concentrations, and side effects. Six databases were searched for articles including adults with BMI ≥35 kg/m^2^ treated with hypocaloric diets before metabolic bariatric surgery. Hypocaloric diets were categorized as (1) low‐energy diet containing 800–1200 kcal/day for 2–4 weeks, (2) very low‐energy diet containing 450–<800 kcal/day for 2–4 weeks, and (3) low‐energy diet containing 800–1200 kcal/day for >4 weeks. Thirty‐three articles (1868 patients) were included, and if data were sufficient, synthesis without meta‐analysis was conducted. A low‐energy diet and very low‐energy diet for 2–4 weeks resulted in similar reductions in total body weight, but longer treatment correlated to a more pronounced weight reduction. In addition, a low‐energy diet for 2–4 weeks led to decreased liver volume, which might facilitate the surgical procedure. Insulin resistance was generally reduced after a low‐energy diet for 2–4 weeks. However, most studies were within‐group control, and since more than 60% of the studies lacked variance measures for our outcomes, we did not perform a meta‐analysis. Accordingly, our results should be interpreted carefully. The protocol was registered in the International Prospective Register of Systematic Reviews (PROSPERO) with registration number: CRD42022295757; available at: https://www.crd.york.ac.uk/prospero/display_record.php?RecordID=295757.

**Abbreviations**
BMI: body mass index
CI: computed tomography
HII: HOMA index IR
HMAIR: homeostatic model assessment of insulin resistance
IQR: interquartile range
Kcal: kilocalories
LED: low‐energy diet
MASLD: metabolic dysfunction‐associated steatotic liver disease
MBS: metabolic bariatric surgery
MRI: magnetic resonance imaging
PROSPERO: population intervention control outcome
TBWL: total body weight loss
VLED: very low‐energy diet

## 1 INTRODUCTION

Obesity is associated with metabolic dysfunction‐associated steatotic liver disease (MASLD) often including an enlarged liver, which can complicate metabolic bariatric surgery (MBS) and make the liver more susceptible to injury.^1^, ^2^ Moreover, perioperative hyperglycemia in MBS patients was associated with a higher risk for infectious complications.^3^ Therefore, treatment with a hypocaloric diet in the preoperative period before MBS is frequently adopted to reduce liver volume and facilitate the surgical procedure.^4^, ^5^ In addition, this type of diet regimen was reported to lower glucose concentrations and improve insulin sensitivity,^6^, ^7^ and two studies reported that preoperative weight loss induced by hypocaloric diets was related to a reduction of postoperative complications after MBS.^8^, ^9^ However, previous systematic reviews and a meta‐analysis found no effect on postoperative complications and stated that the effect of preoperative weight reduction before MBS on postoperative complication risk is still unclear.^10^, ^11^

To date, there is no consensus regarding energy content and duration of hypocaloric diets used before MBS. Low‐energy diets (LED) and very low‐energy diets (VLED) consisting of diet replacement products or food‐based diets are common.^10^, ^12^ Moreover, national surveys of preoperative hypocaloric diet strategies revealed that there are large discrepancies in treatment practice within and between countries.^13^, ^14^, ^15^ Furthermore, excessive caloric restriction might lead to more side effects and larger loss of lean body mass.^4^, ^16^

Thus, to clarify the appropriate energy content and treatment duration, this systematic review aimed to examine the effect of hypocaloric diets in the preoperative period before MBS. Our objectives were to compare the effects of prescribed hypocaloric diets on total body weight loss (TBWL), reduction in liver volume, glucose and insulin concentrations, and side effects (Table 1).

**TABLE 1:** Review question illustrated by PICO.^17^

Parameter	Abbreviation	Inclusion criteria
Population	P	Adult patients accepted for MBS with BMI ≥35 kg/m^2^
Intervention	I	Prescribed LED treatment with short duration (800–1200 kcal/day, 2–4 weeks)
Control	C	Normal diet Prescribed VLED treatment with short duration (450–<800 kcal/day, 2–4 weeks) Prescribed LED treatment with long duration (800–1200 kcal/day, >4 weeks)
Outcome	O	Total body weight loss Reduction in liver volume Reduction in glucose and insulin concentrations Side effects

Abbreviations: BMI, body mass index; LED, low‐energy diet; MBS, metabolic bariatric surgery; PICO, Population Intervention Control Outcome; VLED, very low‐energy diet.

## 2 MATERIALS AND METHODS

### 2.1 Study protocol

We used established guidelines for planning, conducting, and reporting this systematic review.^17^, ^18^, ^19^ The protocol was registered in the International Prospective Register of Systematic Reviews (PROSPERO)^20^ with registration number: CRD42022295757; available at: https://www.crd.york.ac.uk/prospero/display_record.php?RecordID=295757.

### 2.2 Eligibility criteria

We included studies of males and females, ≥18 years old and BMI ≥35 kg/m^2^ treated with a defined hypocaloric diet in the preoperative period before MBS. Studies including other weight‐reducing interventions were excluded. Randomized and nonrandomized controlled studies were included as well as single‐arm studies.

### 2.3 Search strategy

The literature search was conducted from June 22th to 29th, 2021, with an updated search on March 16th, 2023, in PubMed, Ovid Medline, CINAHL, Embase, Global Index Medicus, and Cochrane Library by an information specialist and the first author (IN) (Table S1 shows search terms and results from PubMed). No limiters were included in the literature searches, and IN manually scanned reference lists of systematic reviews and searched for ongoing studies in ClinicalTrials.gov.

### 2.4 Study selection and categorization

Records identified through databases were uploaded to Endnote™ (version X9) and duplicates, articles written in languages other than English, as well as titles deemed ineligible, were removed before transfer of records to Rayyan.^21^ Each of the following steps in conducting the systematic review was performed independently by two authors, and disagreements were solved by consensus. All abstracts were classified in Rayyan as “included,” “excluded,” or “maybe” and when classified as “included” or “maybe,” full text articles were retrieved and reviewed using the eligibility criteria. We documented study details and inclusion or exclusion status in an Excel spreadsheet.

Studies were categorized according to the review question as either between‐group control (intervention vs. control group continuing with their normal diet) or within‐group control (before and after hypocaloric diet in the same patients) (Table 2). For some two‐armed studies, we included only the arm that corresponded to our review question (Table S2).

**TABLE 2:** Number of studies and patients, calorie content, and diet duration categorized according to the review question.

Treatment category	Abbreviation used in systematic review	Control	Number of studies/patients	Energy content, kcal/day^a^	Diet duration, days^a^
LED treatment short duration (800–1200 kcal/day, 2–4 weeks) versus normal diet^7^, ^9^, ^22^, ^23^, ^24^, ^25^	LED short duration versus normal diet	Between‐group control	6/499	900 (800–1150)	28 (18–28)
LED treatment short duration (800–1200 kcal/day, 2–4 weeks)^1^, ^6^, ^26^, ^27^, ^28^, ^29^, ^30^, ^31^, ^32^, ^33^, ^34^, ^35^, ^36^, ^37^	LED short duration	Within‐group control	14/839	858 (800–995)	21 (14–25)
VLED treatment short duration (450–<800 kcal/day, 2–4 weeks)^26^, ^38^, ^39^, ^40^, ^41^, ^42^, ^43^, ^44^, ^45^	VLED short duration	Within‐group control	9/361	636 (601–673)	14 (14–21)
LED treatment long duration (800–1200 kcal/day, >4 weeks)^46^, ^47^, ^48^, ^49^	LED long duration	Within‐group control	4/169	965 (875–1073)	49 (47–51)

Abbreviations: IQR, interquartile range; LED, low‐energy diet; VLED, very low‐energy diet.

^a^ Data are median (IQR).

### 2.5 Data extraction and analysis

A predefined Excel spreadsheet was used for extraction of data on study design, sample size, dropout rate, age, gender, BMI, diabetes, hypocaloric diet description and duration, adherence method, outcomes for total body weight, liver volume, glucose and insulin concentrations, and side effects. If data were missing, we requested additional information from the study authors by mail.

From the extracted data on glucose and insulin concentrations, the homeostatic model assessment of insulin resistance (HOMA index IR) was calculated (P‐insulin × B‐glucose/22.5).^50^

This systematic review did not allow meta‐analysis or meta‐regression due to lack of variance measures for our outcome data in more than 60% of the studies. Hence, we performed a synthesis without meta‐analysis reporting median 25th–75th percentiles (IQR) and minimum‐maximum values (range).^17^, ^18^ Because of asymmetric data, we used Spearman's rho to examine potential correlations between energy content and duration of the hypocaloric diet, the number of study patients, age, baseline BMI, percentage of patients with female gender or with Type 2 diabetes per study on the one hand, and our outcome data on the other hand. Level of significance was *p* < 0.05. Jamovi (version 2.3.28) was used for descriptive data and statistics.

### 2.6 Assessment of risk of bias in individual studies

To evaluate the risk of bias for individual studies, we used the National Heart, Lung and Blood Institute's appraisal tools for controlled intervention studies and before‐after studies.^51^ The appraisal tool for controlled intervention studies consisted of 14 items of which Items 1, 5, 6, 7, 8, and 12 were given greater weight than the remaining items (Table S3). The tool for before‐after studies consisted of 12 items, but Item 12 was excluded since it was not applicable, and Items 1, 5, 7, 8, 9, and 10 were given greater weight (Table S4). Furthermore, we added one item regarding conflict of interest to both tools. Each item was evaluated in relation to the outcomes for this review and answered with yes, no, or other and with an overall risk of bias assessment for each study as good, fair, or poor (Tables S3 and S4).

## 3 RESULTS

### 3.1 Article selection, study characteristics, and categorization

Of 2341 identified records, we assessed 140 full text articles for eligibility of which 33 (reporting results from 32 studies) were included in the systematic review (Flow diagram Figure S1). Categorization and characteristics of the studies included are shown in Tables 2, S2, and S5. Six studies were categorized as between‐group control studies of which four were randomized controlled studies. Twenty‐six studies were categorized as within‐group control studies including six controlled studies that were treated as within‐group control since only one of the arms was part of our inclusion criteria or review question. Furthermore, six of the studies classified as within‐group control included two different hypocaloric diets that were categorized in the same treatment category (five studies) or in different treatment categories (one study).

The 32 studies included a total of 1868 patients (69% female) with a median (IQR, range) baseline BMI, age, and sample size per study of 45.3 kg/m^2^ (42.8–47.4, 39.4–57.2), 43 years^32^, ^33^, ^34^, ^35^, ^36^, ^37^, ^38^, ^39^, ^40^, ^41^, ^42^, ^43^, ^44^, ^45^, ^46^, ^47^, ^48^, ^49^ and 34 patients (19–41, 7–305), respectively. Median BMI, age, and sample size were similar between the study categories (Table S6). Moreover, all but four studies had mean or median baseline BMI levels below 50 kg/m^2^ (Table S5).

### 3.2 Outcome results

#### 3.2.1 TBWL

**FIGURE 1:**
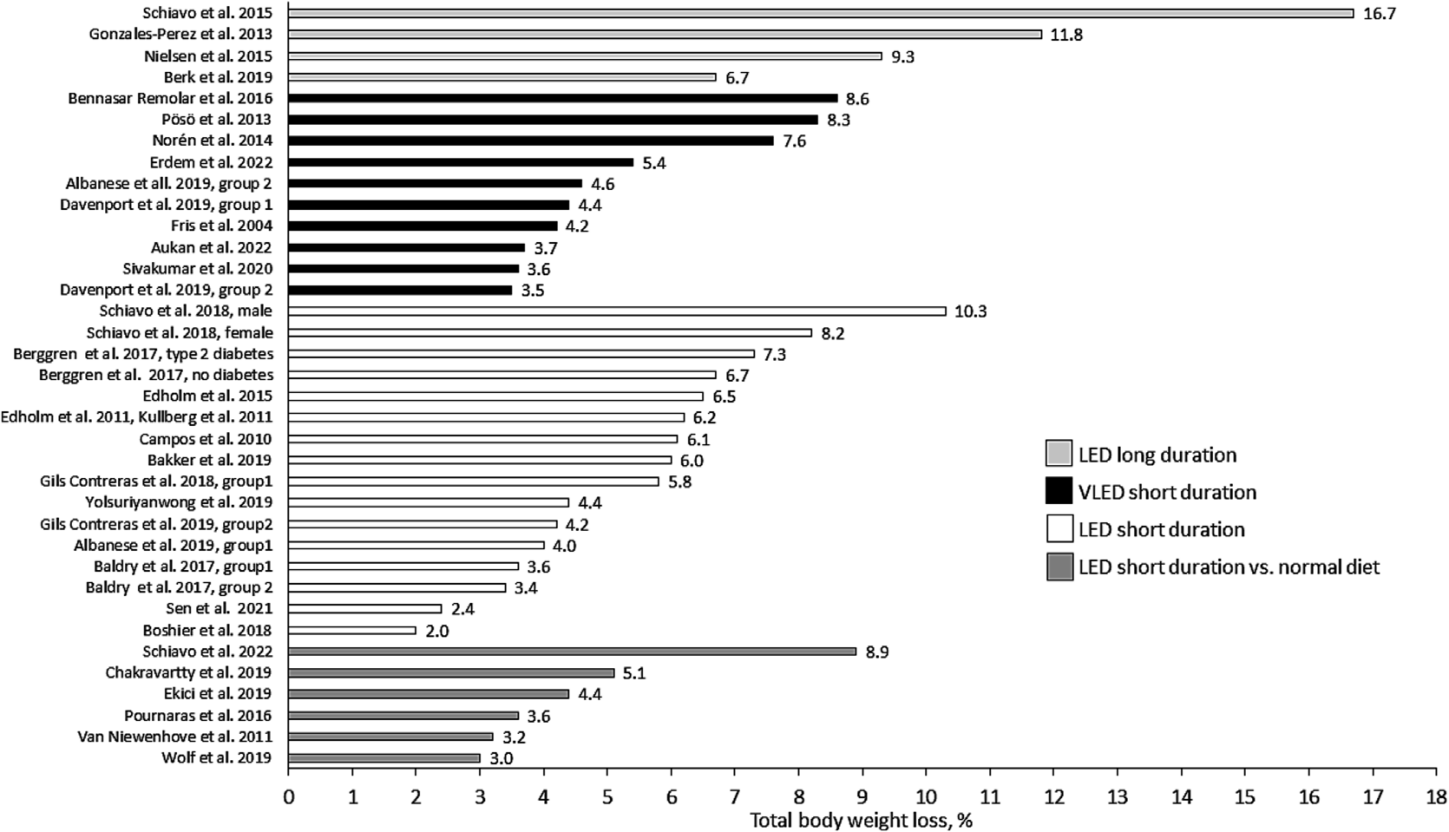
Percentage total body weight loss after treatment with hypocaloric diets in 30 studies (36 patient groups). LED short duration versus normal diet where LED short duration is 800–1200 kcal/day, 2–4 weeks; VLED short duration is 450–<800 kcal/day, 2–4 weeks; LED long duration is 800–1200 kcal/day, >4 weeks. LED, low‐energy diet; VLED, very low‐energy diet.

Thirty‐two studies reported data on TBWL (Table 3 and Figure 1). For two studies with low overall risk of bias, patients were treated with 800 kcal/day of powder‐based diets for 14 and 28 days, which resulted in a TBWL of 3.2% and 3.6%.^7^, ^9^ Moreover, three studies demonstrated among the largest TBWL, 8.2%—16.7% (Figure 1).^24^, ^35^, ^49^ These studies used 2–4 quantified hypocaloric food‐based meal plans with adherence checked repeatedly with validated dietary assessment methods throughout the study period. Two other studies reported the lowest TBWL of 2.0% and 2.4%, respectively.^30^, ^36^ Both studies reported energy content and duration of LED but otherwise with sparse dietary information about the hypocaloric diet intervention.

**TABLE 3:** Description of hypocaloric diet, total body weight at baseline, and TBWL after treatment with hypocaloric diet for 32 studies (39 patient groups).

Author, publication year	Treatment category	Type of diet	Energy content in hypocaloric diet, kcal/day	Diet duration, days	Total body weight at baseline, kg	TBWL after hypocaloric diet, kg	TBWL after hypocaloric diet, %
Chakravartty et al., 2019	LED short duration versus normal diet	Food‐based/normal diet	800	28	125^a^/135.9^a^	6.7^a^/0.4^a^	5.4/0.3
Ekici et al., 2019	LED short duration versus normal diet	Food‐based/normal diet	1000	28	124.4 ± 14.1/124.7 ± 14.1	5.5 ± 1.5/0	4.4/0
Pournaras et al., 2016	LED short duration versus normal diet	Powder‐based/normal diet	800	28	116.9 ± 13.2/129 ± 24.8	4.8/0.7	4.1/0.5
Schiavo et al., 2022	LED short duration versus normal diet	Food‐ and powder‐based/normal diet	1200	28	143.6 ± 23.6/132.7 ± 23.0	13.9/1.1	9.7/0.8
Van Niewenhove et al., 2011	LED short duration versus normal diet	Powder‐based/normal diet	800	14	130.3 ± 23.7/127 ± 22.8	4.9/0.4	3.5/0.3
Wolf et al., 2019	LED short duration versus normal diet	Food‐ and powder‐based/normal diet	1200	14	128.6 ± 25.4/136.10 ± 24.1	3.4/+0.6	2.6/+0.4
Albanese et al., 2019, Group 1	LED short duration	Food‐based	800	21	120.9 ± 22.6	4.8 ± 2.5	4.0
Bakker et al., 2019	LED short duration	Powder‐based	800	14	116.9 ± 17.4	6.5 ± 4.5	6.0 ± 4.0
Baldry et al., 2017, Group 1	LED short duration	Food‐based	800	14	NR	4.7^a^ (group 1 + 2)	3.6
Baldry et al., 2017, Group 2	LED short duration	Powder‐based	800	14	NR		3.4
Berggren et al., 2017, Type 2 diabetes	LED short duration	Powder‐based	858	25	111.6 ± 13.6	8.2	7.3
Berggren et al., 2017, no diabetes	LED short duration	Powder‐based	858	25	114.2 ± 11.1	7.7	6.7
Boshier et al., 2018	LED short duration	Food‐based	900	21	124.7 ± 25.3	2.5	2.0
Campos et al., 2010	LED short duration	Powder‐based	800	14	134.7 ± 16.9	8.2 ± 2.3	6.1
Cleveland et al., 2016	LED short duration	Food‐ and powder‐based	1000	14	NR	2.4	NR
Edholm et al., 2011, Kullberg et al., 2011	LED short duration	Powder‐based	959	28	121.3 ± 13.4	7.5 ± 2.6	6.2
Edholm et al., 2015	LED short duration	Powder‐based	990	28	114.3 ± 12.1	7.4 ± 1.2	6.5
Gils Contreras et al., 2018, Group 1	LED short duration	Powder‐based	800	21	131.9 ± 22.6	7.7 ± 2.7	5.8
Gils Contreras et al., 2018, Group 2	LED short duration	Food‐ and powder‐based	1200	21	126.2 ± 17.1	5.4 ± 2.2	4.2
Lange et al., 2022, Group 1	LED short duration	Powder‐based	913	14	NR	5.2 (group 1 + 2)	NR
Lange et al., 2022, Group 2	LED short duration	Powder‐based	841	14	NR		NR
Schiavo et al., 2018, male	LED short duration	Food‐based	1200	28	136.3 ± 6.7	14.1	10.3
Schiavo et al., 2018, female	LED short duration	Food‐based	1200	28	127.5 ± 5.0	10.4	8.2
Sen et al., 2021	LED short duration	Powder‐based	1000	14	125.6 ± 28.4	3.0	2.4
Yolsuriyanwong et al., 2019	LED short duration	Powder‐based	800	14	160.3 ± 20.1	7.0 ± 2.3	4.4
Albanese et al., 2019, Group 2	VLED short duration	Food‐ and powder‐based	700	21	125.5 ± 19.5	5.8 ± 2.4	4.6
Aukan et al., 2022	VLED short duration	Powder‐based	750	14	119.0 (112.0–126.0)*	4.4 (2.8–5.9)*	3.7
Bennasar Remolar et al., 2016	VLED short duration	Powder‐based	603	28	129.8 ± 26.0	11.1	8.6
Davenport et al., 2019, Group 1	VLED short duration	Powder‐based	636	14	119.9 ± 21.7	5.5	4.4 ± 2.3
Davenport et al., 2019, Group 2	VLED short duration	Powder‐based	636	14	120.4 ± 20.5	4.2	3.5 ± 1.9
Erdem et al., 2022	VLED short duration	Powder‐based	650	14	125.3 (48)^a^	6.4 (3.2)^a^	5.4
Fris et al., 2004	VLED short duration	Powder‐based	456	14	125 ± 20.4	5.2 (4.4–6.0)*	4.2
Norén et al., 2014	VLED short duration	Powder‐based	680	28	107.4 (99.6–115.1)*	8.2 (7.1–9.2)*	7.6
Pösö et al., 2013	VLED short duration	Powder‐based	600	21	NR	11.3 ± 3.6	8.3 ± 1.9
Sivakumar et al., 2020	VLED short duration	Powder‐based	600	14	121.6 ± 18.9	4.5 ± 2.3	3.6 ± 1.6
Berk et al., 2019	LED long duration	Food‐based	900	49	137.1 ± 21.9	9.2	6.7
Gonzales‐Perez et al., 2013	LED long duration	Food‐based	800	42	122.5^a^ ± 12.6	14.5^a^	11.8
Nielsen et al., 2015	LED long duration	Food‐ and powder‐based	1030	49	135.1 ± 19.2	12.7 ± 4.4	9.3 ± 0.5
Schiavo et al., 2015	LED long duration	Food‐based	1200	56	141 ± 9.6	23.6	16.7

*Note*: Data are mean ± SD. TBWL is mean difference (reported by studies) or difference in means (computed by review team, that is, difference in pre‐ to post means). **Powder‐based diets:** Modifast, Optifast, Optifast HP, Lighter Life, Atkins shake, BCM Diät, SLANKA, Very low‐calorie ketogenic diet, Formulite, Allevo, Nutrilett, Naturdiet. Some studies allowed low starch vegetables. **Food‐based diets:** Cambridge milk diet, diet plans consisting of normal food, Derby Teaching Hospitals NHS Foundation Trust standard prebariatric surgery food‐based diet, low‐carbohydrate diet, Mediterranean diet. **Food‐ and powder‐based diets:** Combination of normal food and protein shakes or Optifast or Ketocompleat, normal food‐based low‐carbohydrate diet and protein shakes or whey protein powder enriched with amino acids, Cambridge Weight Plan consisting of powder‐based meals and skimmed milk, vegetables, and low‐fat yoghurt.

Abbreviations: CI, confidence interval; IQR, interquartile range; LED, low‐energy diet; NR, not reported; SD, standard deviation; TBWL, total body weight loss; VLED, very low‐energy diet.

^a^ median (IQR).

* 95%CI.

**FIGURE 2:**
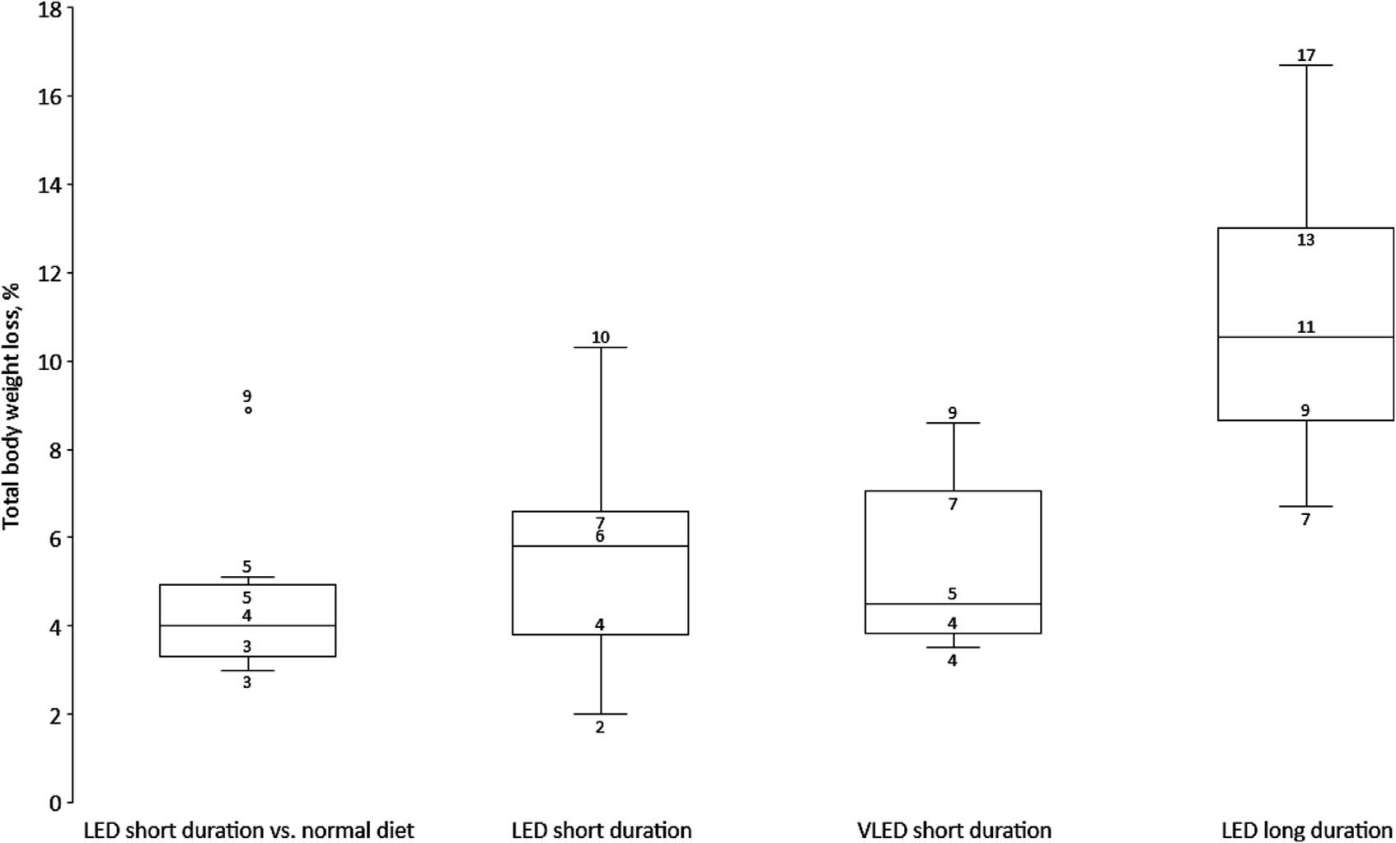
Box and whiskers plot for percentage total body weight loss for each treatment category. LED short duration versus normal diet: six studies. LED short duration: 12 studies (16 patient groups). VLED short duration: nine studies (10 patient groups). LED long duration: four studies. Data are median, 25th–75th percentiles, minimum‐maximum values and outliers. LED short duration versus normal diet where LED short duration is 800–1200 kcal/day, 2–4 weeks. VLED short duration is 450–<800 kcal/day, 2–4 weeks. LED long duration is 800–1200 kcal/day, >4 weeks. LED, low‐energy diet; VLED, very low‐energy diet.

The median (IQR, range) TBWL for LED short duration versus normal diet was 5.0 kg (4.2–6.1, 4.0–12.8), LED short duration was 7.0 kg (4.8–7.7, 2.4–14.1), VLED short duration was 5.7 kg (4.7–7.8, 4.2–11.3), and LED long duration was 13.6 kg (11.8–16.8, 9.2–23.6) with percentages shown in Figure 2.

**FIGURE 3:**
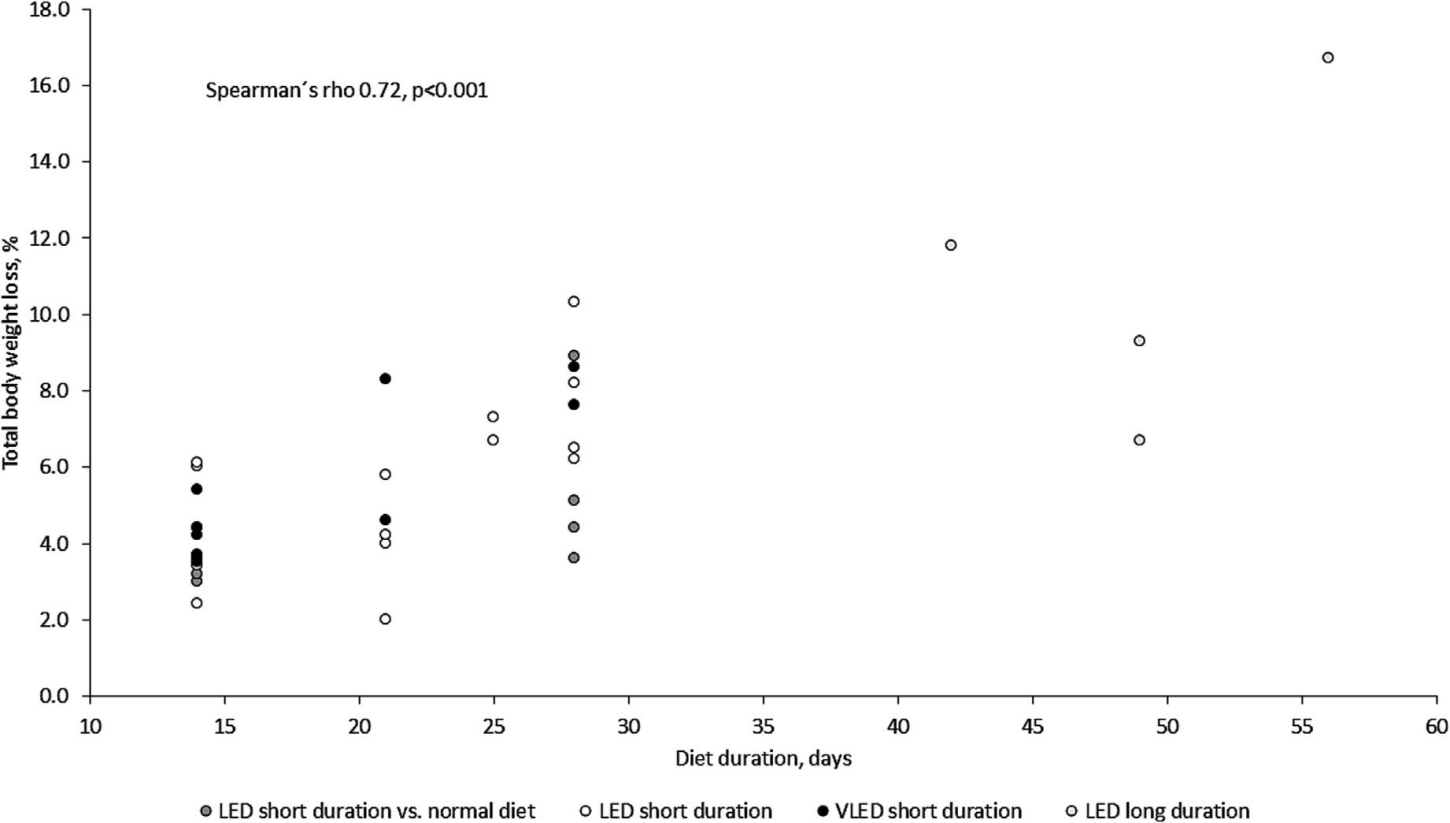
Correlation between hypocaloric diet duration and percentage total body weight loss. LED short duration versus normal diet: six studies. LED short duration: 12 studies (16 patient groups). VLED short duration: nine studies (10 patient groups), and LED long duration: four studies. LED short duration versus normal diet where LED short duration is 800–1200 kcal/day, 2–4 weeks. VLED short duration is 450–<800 kcal/day, 2–4 weeks. LED long duration is 800–1200 kcal/day, >4 weeks. LED, low‐energy diet; TBWL, total body weight loss; VLED, very low‐energy diet.

We found a significant positive correlation between diet duration and TBWL% when including all studies (Figure 3) and when omitting studies using VLED short duration (0.73, *p* < 0.001) or LED long duration (0.62, *p* < 0.001). However, we found no significant correlations between the energy content and TBWL%, either between the number of study patients, age, baseline BMI, percentage of patients with female gender, or with Type 2 diabetes per study on the one hand and TBWL% on the other (Spearman's rho<0.3, *p* > 0.1).

#### 3.2.2 Reduction in liver volume

**FIGURE 4:**
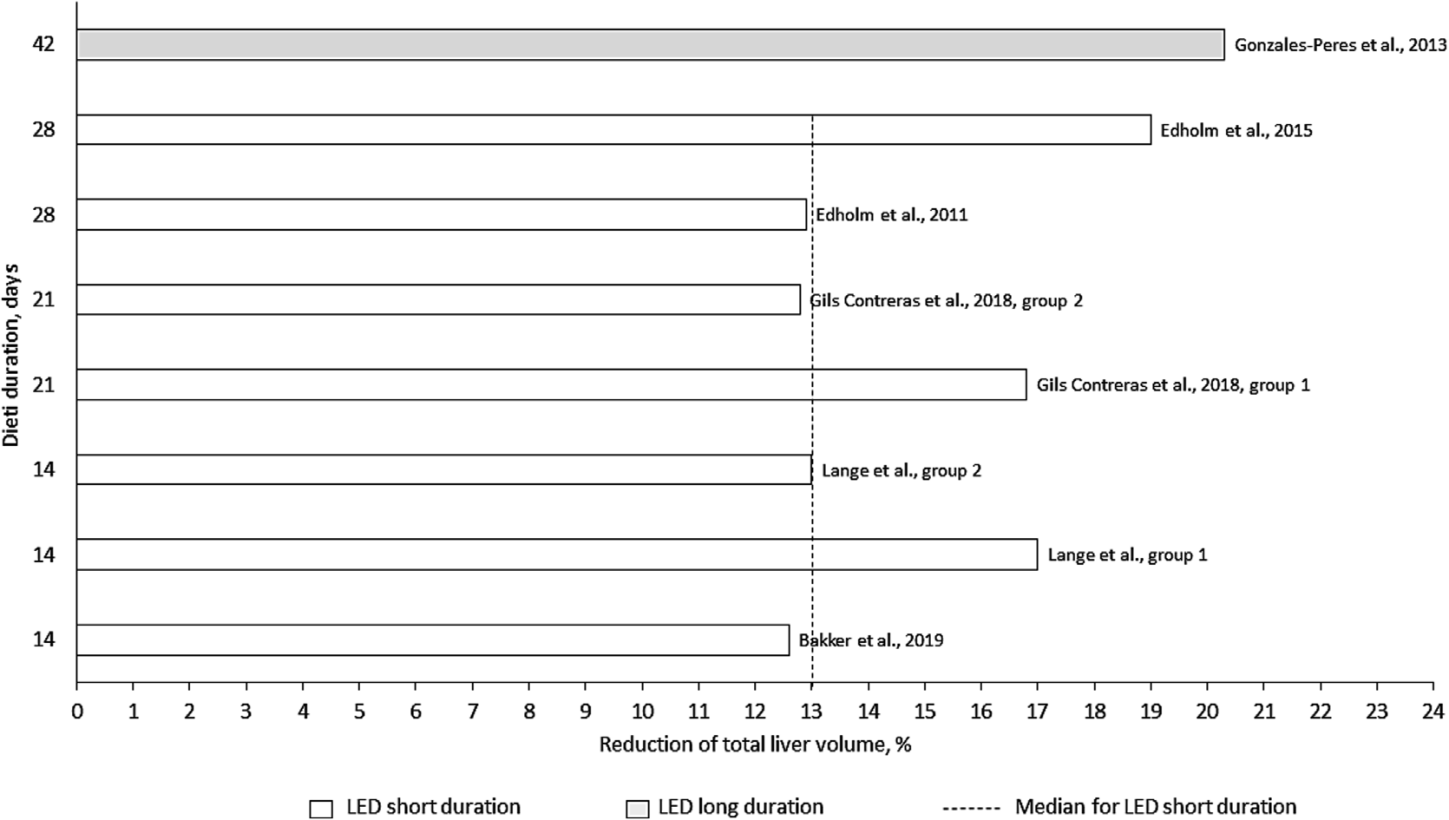
Reduction in total liver volume and diet duration for studies using LED with short (five studies, seven patient groups) and long duration (one study). LED short duration is 800–1200 kcal/day, 2–4 weeks. LED long duration is 800–1200 kcal/day, >4 weeks. LED, low‐energy diet; VLED, very low‐energy diet.

Eight studies measured total liver volume using computed tomography (CT) or magnetic resonance imaging (MRI) or volume of the left liver lobe using ultrasound (Table 4). Figure 4 shows reduction in total liver volume and diet duration.

**TABLE 4:** Liver volume at baseline and reduction after hypocaloric diet for eight studies (10 patient groups).

Author, publication year	Treatment category	Method for assessment of liver volume	Mean liver volume at baseline, mL	Reduction in liver volume after diet, mL	Reduction in liver volume after diet, %
Bakker et al., 2019	LED short duration	Total liver, MRI	2137 (553)^a^	269 (287)^a^	12.6
Edholm et al., 2011	LED short duration	Total liver, MRI	2170 ± 370	280	12.9
Edholm et al., 2015	LED short duration	Total liver, MRI	2100 ± 700	400	19.0
Lange et al., 2022, Group 1	LED short duration	Total liver, MRI	2687 ± 691	457 (349–565)*	17.0
Lange et al., 2022, Group 2	LED short duration	Total liver, MRI	2634 ± 576	342 (255–429)*	13.0
Gils Contreras et al., 2018, Group 1	LED short duration	Total liver, CT	2653 ± 654	445 ± 432	16.8
Gils Contreras et al., 2018, Group 2	LED short duration	Total liver, CT	2600 ± 833	332 ± 340	12.8
Gonzales‐Perez et al., 2013	LED long duration	Total liver, CT	2295^a^	465^a^	20.3
Chakravartty et al., 2019	LED short duration versus normal diet	Left liver lobe, Ultrasound	403^a^/435^a^	93/9^a^	23.0/2.1
Bakker et al., 2019	LED short duration	Left liver lobe, Ultrasound	593 (182)^a^	104 (139)^a^	17.5
Schiavo et al., 2018	LED short duration	Left liver lobe, Ultrasound	627 ± 85	124	19.8

*Note*: Data are mean ± SD. Reduction in liver volume is mean difference (reported by studies) or difference in means (computed by review team, i.e., difference in pre‐ to post means). LED short duration versus normal diet where LED short duration is 800–1200 kcal/day, 2–4 weeks. VLED short duration is 450–<800 kcal/day, 2–4 weeks. LED long duration is 800–1200 kcal/day, >4 weeks.

Abbreviations: CI, confidence interval; CT, computed tomography; IQR, interquartile range; LED, low‐energy diet; MRI, magnetic resonance imaging; SD, standard deviation; VLED, very low‐energy diet.

^a^ median (IQR).

* 95%CI.

The median (IQR, range) total liver volume at baseline for studies using LED short duration was 2600 mL (2154–2644, 2100–2687), and the reduction in total liver volume after LED treatment was 342 mL (306–423, 269–457) or 13%.^13^, ^14^, ^15^, ^16^, ^17^, ^18^, ^19^ No data synthesis was possible for the remaining study categories, and no correlation estimates were performed between TBWL% and reduction in total liver volume since only five studies reported data for both outcomes.

#### 3.2.3 Reduction in fasting glucose and insulin concentrations and insulin resistance

Fasting glucose and/or insulin concentrations were reported by 13 studies (Table 5).

**TABLE 5:** Fasting glucose and insulin concentrations and HOMA index IR at baseline and reductions after hypocaloric diet for 13 studies (15 patient groups).

Author, publication year	Treatment category	Type 2 diabetes, %	Glucose conc at baseline, mmol/L	Reduction in glucose conc, mmol/L	Reduction in glucose conc, %	Insulin conc at baseline, mIU/L	Reduction in insulin conc, mIU/L	Reduction in insulin conc, %	HOMA index IR at baseline	Reduction in HOMA index IR, %
Pournaras et al., 2016	LED short duration versus normal diet	100/0	9.9 ± 3.7/ 6.8 ± 2.2	3.2/0.6	32/9	26.7 ± 17.7/22.3 ± 10.5	13.2/0.1	49.4/0.4	11.8/6.7	66/9
Schiavo et al., 2022	LED short duration versus normal diet	NR	NR	NR	NR	11.8 ± 6.3/11.0 ± 7.0	1.2/0.6	10.2/5.5	NR	NR
Berggren et al., 2017, Group 1	LED short duration	100	7.3 ± 3.8	0.8	11	25.0 ± 18.9	6.3	25.2	8.1	33
Berggren et al., 2017, Group 2	LED short duration	0	5.1 ± 1.2	0.0	0	23.2 ± 14.1	7.2	31	5.3	32
Campos et al., 2010	LED short duration	0	5.5 ± 0.8	0.7 ± 1.0	12.7	34.1 ± 20.1	13.7 ± 15.9	40.2	8.3	47
Edholm et al., 2015	LED short duration	NR	5.8 ± 0.6	0.1	1.7	21.9 ± 9.4	4.5	20.5	5.7	23
Gils Contreras et al., 2018, Group 1	LED short duration	25.6	6.8 ± 2.8	1.4 ± 2.5	20.6	23.2 ± 14.1	8.2 ± 10.5	35.3	7.0	49
Gils Contreras et al., 2018, Group 2	LED short duration	9.8	5.7 ± 1.9	0.4 ± 1.1	7.0	19.9 ± 10.5	4.0 ± 6.5	20.1	5.0	24
Kullberg et al., 2011	LED short duration	NR	5.1 ± 0.7	0.4	7.8	22.9 ± 7.8	5.8	25.3	5.2	31
Schiavo et al., 2018	LED short duration	22.2	6.4 ± 1.5	1.7	26.6	10.5 ± 5.9	3.9	37.1	3.0	53
Erdem et al., 2022	VLED short duration	NR	6.7 (6.6)^a^	0.8 (2.7)^a^	11.9	NR	NR	NR	NR	NR
Norén et al., 2014	VLED short duration	28	5.6 (1.5)^a^	0.8^a^	14.3	NR	NR	NR	NR	NR
Berk et al., 2022	LED long duration	29.3	5.4 (1.2)^a^	0.3^a^	5.6	NR	NR	NR	NR	NR
Nielsen et al., 2015	LED long duration	0	5.9 ± 1.1	0.5 ± 0.6	8.5	19.0 ± 13.1	7.4 ± 11.0	38.9	5.0	44
Schiavo et al., 2015	LED long duration	NR	6.6 ± 1.0	1.5	22.4	21.3 ± 5.9	12.4	58.2	6.3	68

*Note*: Data are mean ± SD. Reduction in fasting glucose and insulin concentrations is mean difference (reported by studies) or difference in means (computed by review team, i.e., difference in pre‐ to post means). LED short duration versus normal diet where LED short duration is 800–1200 kcal/day, 2–4 weeks. VLED short duration is 450–<800 kcal/day, 2–4 weeks. LED long duration is 800–1200 kcal/day, >4 weeks. HOMA index IR was computed by the review team (P‐insulin × B‐glucose/22.5).

Abbreviations: Conc, concentration; HOMA index IR, homeostatic model assessment of insulin resistance; IQR, interquartile range; LED, low‐energy diet; NR, not reported; SD, standard deviation; VLED, very low‐energy diet.

^a^ median (IQR).

The median (IQR, range) fasting glucose concentrations at baseline for studies using LED short duration was 5.8 mmol/L (5.4–6.5, 5.1–7.3), and the reduction in fasting glucose concentration after LED treatment was 0.6 mmol/L (0.3–1.0, 0–1.7) or 9% (6–15, 0–27). In addition, in studies using LED short duration, the baseline fasting insulin concentration was 23 mIU/L (21.4–23.6, 10.5–34.1), and the reduction in fasting insulin concentration after LED was 6.1 mIU/L (4.4–7.5, 3.9–13.7) corresponding to 28%.^20^, ^21^, ^22^, ^23^, ^24^, ^25^, ^26^, ^27^, ^28^, ^29^, ^30^, ^31^, ^32^, ^33^, ^34^, ^35^, ^36^, ^37^, ^38^, ^50^, ^51^ Moreover, the average reduction of insulin resistance calculated as HOMA index IR was 33% for LED short duration. No data synthesis was possible for the remaining study categories because of a limited number of studies. Furthermore, we found no significant correlation between TBWL% and reduction in fasting glucose (13 studies, 14 patient groups) or insulin concentrations (11 studies, 12 patient groups) (Spearman's rho<0.2, *p* > 0.6).

#### 3.2.4 Side effects

Eleven studies reported data on side effects. Of these, seven studies used short‐duration LED,^6^, ^27^, ^28^, ^33^, ^34^, ^35^, ^37^ one study used short‐duration VLED,^40^ and three studies used long‐duration LED.^47^, ^48^, ^49^ Side effects were examined by different questionnaires, interviews. or methods not described. Results were reported as percentage of patients having side effects^6^, ^27^, ^33^, ^34^, ^48^ or graded on scales having alternatives such as always, sometimes, occasionally or never.^28^, ^35^, ^37^, ^47^, ^49^ No synthesis of side‐effect data was possible due to the large methodological variation.

Hunger was reported as sometimes or frequently by 15%–71% of the patients.^27^, ^28^, ^35^, ^37^, ^47^, ^49^ Furthermore, headache was reported by 6%–57% of patients,^34^, ^35^, ^48^, ^49^ constipation by 15%–43%,^27^, ^34^, ^35^, ^48^ and other gastrointestinal symptoms such as abdominal pain, flatulence, and loose stool were reported by 10%–19% of the patients.^27^, ^33^, ^34^, ^47^, ^49^ Dizziness or fatigue were reported by 0%–50%^6^, ^34^, ^48^ and halitosis by 28%–100% of patients.^27^, ^35^

### 3.3 Risk of bias for individual studies

Two randomized controlled studies were assessed as good, three controlled studies as fair and one as poor (Table 6). Twenty‐two within‐group control studies were evaluated as fair, three as fair or poor, and two as poor (Table 7). None of the controlled studies and 32% of the within‐group control studies reported data on adherence using validated dietary assessment methods. Two controlled and seven within‐group control studies had risk of bias due to missing data since loss to follow‐up was >20% or the studies did not report loss to follow‐up. In addition, the people who assessed outcomes were not blinded for the patients' group assignments in three out of six controlled and in most of the within‐group control studies. Finally, about one third of the studies reported that commercial companies provided the hypocaloric diets and accordingly conflict of interest could not be excluded (Tables 6 and 7).

**TABLE 6:** Risk of bias for the controlled intervention (between‐group control) studies.

Authors	Treatment category	1	2	3	4	5	6	7	8	9	10	11	12	13	14	15	Rating
Pournaras et al., 2016	LED short duration versus normal diet	**Y**	**Y**	**Y**	**N**	**Y**	**N**	**Y**	**Y**	**NR**	**NR**	**Y**	**NR**	**Y**	**Y**	**Y**	**Good**
Van Nieuwenhove et al., 2011	LED short duration versus normal diet	**Y**	**Y**	**Y**	**N**	**Y**	**Y**	**Y**	**Y**	**NR**	**NR**	**Y**	**NR**	**Y**	**N**	**CD**	**Good**
Chakravartty et al., 2019	LED short duration versus normal diet	**Y**	**Y**	**Y**	**N**	**Y**	**Y**	**N**	**N**	**NR**	**Y**	**Y**	**NR**	**Y**	**N**	**Y**	**Fair**
Ekici et al., 2019	LED short duration versus normal diet	**N**	**N**	**N**	**N**	**N**	**Y**	**N**	**NR**	**NR**	**NR**	**Y**	**NR**	**Y**	**N**	**Y**	**Poor**
Wolf et al., 2019	LED short duration versus normal diet	**N**	**N**	**N**	**N**	**N**	**Y**	**Y**	**Y**	**NR**	**Y**	**Y**	**NR**	**Y**	**Y**	**Y**	**Fair**
Schiavo et al., 2022	LED short duration versus normal diet	**Y**	**Y**	**Y**	**N**	**N**	**NR**	**Y**	**Y**	**NR**	**Y**	**Y**	**NR**	**Y**	**N**	**CD**	**Fair**

*Note*: Items: 1. Randomized controlled study? 2. Adequate randomization method? 3. Concealed treatment allocation? 4. Participants and providers blinded? 5. People assessing outcomes blinded? 6. Groups similar at baseline? 7. Dropout‐rate 20% or lower? 8. 15% or lower difference in dropout rate between groups? 9. High adherence to intervention? 10. Other interventions avoided? 11. Outcome measures valid, reliable, and assessed consistently? 12. Sufficient power? 13. Predefined outcomes and subgroup analyses? 14. Intention‐to‐treat analyses? 15. Conflict of interest? The complete questions are shown in Table S3.

Abbreviations: CD, cannot determine; N, no; NR, not reported; Y, yes.

**TABLE 7:** Risk of bias for the before and after (within‐group control) studies.

Authors	Treatment category	1	2	3	4	5	6	7	8	9	10	11	12	Rating
Albanese et al., 2019	LED short duration	**Y**	**Y**	**Y**	**Y**	**NR**	**NR**	**Y**	**NR**	**Y**	**Y**	**N**	**Y**	**Fair**
Bakker et al., 2019	LED short duration	**Y**	**Y**	**Y**	**N**	**NR**	**Y**	**Y**	**Y**	**Y**	**Y**	**N**	**Y**	**Fair**
Baldry et al., 2017	LED short duration	**Y**	**Y**	**Y**	**N**	**NR**	**Y**	**Y**	**N**	**Y**	**Y**	**N**	**CD**	**Fair**
Berggren et al., 2017	LED short duration	**Y**	**Y**	**Y**	**NR**	**NR**	**NR**	**Y**	**NR**	**NR**	**Y**	**N**	**Y**	**Fair‐Poor**
Boshier et al., 2018	LED short duration	**Y**	**Y**	**Y**	**NR**	**NR**	**NR**	**Y**	**NR**	**Y**	**Y**	**N**	**Y**	**Fair**
Campos et al., 2010	LED short duration	**Y**	**Y**	**Y**	**NR**	**NR**	**NR**	**Y**	**NR**	**N**	**Y**	**N**	**Y**	**Fair‐Poor**
Cleveland et al., 2016	LED short duration	**Y**	**N**	**Y**	**NR**	**NR**	**Y**	**Y**	**NR**	**Y**	**Y**	**N**	**Y**	**Fair**
Edholm et al., 2011	LED short duration	**Y**	**Y**	**Y**	**Y**	**NR**	**NR**	**Y**	**N**	**Y**	**Y**	**N**	**CD**	**Fair**
Edholm et al., 2015	LED short duration	**Y**	**Y**	**Y**	**N**	**NR**	**NR**	**Y**	**Y**	**Y**	**Y**	**N**	**CD**	**Fair**
Gils Contreras et al., 2018	LED short duration	**Y**	**Y**	**Y**	**N**	**Y**	**Y**	**Y**	**NR**	**Y**	**Y**	**N**	**CD**	**Fair**
Lange et al., 2022	LED short duration	**Y**	**Y**	**Y**	**Y**	**Y**	**Y**	**Y**	**NR**	**N**	**Y**	**N**	**Y**	**Fair**
Schiavo et al., 2018	LED short duration	**Y**	**Y**	**Y**	**NR**	**NR**	**Y**	**Y**	**NR**	**Y**	**Y**	**N**	**CD**	**Fair**
Sen et al., 2021	LED short duration	**Y**	**Y**	**Y**	**Y**	**NR**	**NR**	**N**	**NR**	**Y**	**N**	**N**	**Y**	**Poor**
Yolsurianwong et al., 2019	LED short duration	**Y**	**Y**	**Y**	**Y**	**NR**	**Y**	**Y**	**NR**	**Y**	**Y**	**N**	**Y**	**Fair**
Albanese et al., 2019	VLED short duration	**Y**	**Y**	**Y**	**Y**	**NR**	**NR**	**Y**	**NR**	**Y**	**Y**	**N**	**Y**	**Fair**
Aukan et al., 2022	VLED short duration	**Y**	**Y**	**Y**	**N**	**NR**	**NR**	**N**	**NR**	**N**	**Y**	**N**	**Y**	**Poor**
Bennasar R. et al., 2016	VLED short duration	**Y**	**Y**	**Y**	**Y**	**NR**	**NR**	**Y**	**NR**	**Y**	**N**	**N**	**Y**	**Fair**
Davenport et al., 2019	VLED short duration	**Y**	**Y**	**Y**	**NR**	**NR**	**NR**	**Y**	**Y**	**Y**	**Y**	**N**	**CD**	**Fair**
Erdem et al., 2022	VLED short duration	**Y**	**Y**	**Y**	**NR**	**Y**	**NR**	**Y**	**NR**	**N**	**Y**	**N**	**Y**	**Fair**
Fris et al., 2004	VLED short duration	**Y**	**Y**	**Y**	**Y**	**NR**	**NR**	**Y**	**Y**	**Y**	**Y**	**N**	**Y**	**Fair**
Norén et al., 2014	VLED short duration	**Y**	**Y**	**Y**	**Y**	**NR**	**NR**	**Y**	**NR**	**Y**	**Y**	**N**	**CD**	**Fair**
Pösö et al., 2013	VLED short duration	**Y**	**N**	**Y**	**Y**	**NR**	**NR**	**Y**	**N**	**Y**	**N**	**N**	**Y**	**Fair**
Sivakumar et al., 2020	VLED short duration	**Y**	**Y**	**Y**	**NR**	**NR**	**NR**	**Y**	**NR**	**N**	**Y**	**N**	**Y**	**Fair‐Poor**
Berk et al., 2022	LED long duration	**Y**	**Y**	**Y**	**N**	**NR**	**NR**	**Y**	**NR**	**Y**	**Y**	**N**	**Y**	**Fair**
Gonzales‐Perez et al., 2013	LED long duration	**Y**	**Y**	**Y**	**NR**	**NR**	**NR**	**Y**	**NR**	**Y**	**Y**	**N**	**Y**	**Fair**
Nielsen et al., 2016	LED long duration	**Y**	**Y**	**Y**	**NR**	**Y**	**Y**	**Y**	**NR**	**Y**	**Y**	**N**	**CD**	**Fair**
Schiavo et al., 2015	LED long duration	**Y**	**Y**	**Y**	**N**	**NR**	**Y**	**Y**	**NR**	**NR**	**Y**	**N**	**Y**	**Fair**

*Note*: Items: 1. Study question clearly stated? 2. Eligibility criteria clearly described? 3. Participants representative for the population of interest? 4. All eligible participants included? 5. Sufficient sample size? 6. Intervention clearly described and delivered consistently? 7. Outcomes pre‐specified, defined, valid, reliable, and assessed consistently? 8. People assessing outcomes blinded? 9. Dropout rate 20% or less? 10. Statistics examined pre‐to‐post changes? 11. Outcomes taken multiple times? 12. Conflict of interest? The complete questions are shown in Table S4.

Abbreviations: CD, cannot determine; LED, low‐energy diets; N, no, NR, not reported; Y, Yes.

## 4 DISCUSSION

This systematic review aimed to explore the effect of different hypocaloric diets used in the preoperative period before MBS to clarify the appropriate energy content and diet duration. Studies using short‐duration LED showed similar TBWL as studies using short‐duration VLED, while long‐duration LED indicated a more pronounced TBWL. Bringing data for all studies together, we found a significant positive correlation between diet duration per study and TBWL%, but not with the energy content of the hypocaloric diet.

Different studies showed a great variation in the average TBWL and only a third of the studies reported adherence using validated dietary assessment methods. As mentioned in Section 3, three studies demonstrating among the largest TBWL, used quantified hypocaloric meal plans and frequently monitored adherence throughout the study period.^24^, ^35^, ^49^ Another important aspect is the preoperative nutritional status since hypoalbuminemia and micronutrient deficiencies have been reported before MBS in earlier studies.^52^, ^53^ Furthermore, a larger degree of caloric restriction might result in greater loss of lean body mass.^16^, ^54^ Consequently, it is important that patients receive adequate support during the hypocaloric diet, undergoes a preoperative nutritional assessment, and that the hypocaloric diet contain sufficient macro‐ and micronutrients.^4^, ^12^

Synthesizing results from individual studies on total liver volume, glucose, and insulin concentrations was possible only for the study category of short‐duration LED. Hence, for short‐duration LED, the median total liver volume was decreased by 13%, from 2600 mL at baseline to 2260 mL, which could be compared with the mean liver volume of 1400–1800 mL in adults without obesity.^55^ However, a clinically meaningful reduction of liver volume in the context of preoperative optimization of patients accepted for MBS has not yet been defined. It has been reported that 4 weeks of treatment with LED improved the exposure of the hiatal region and resulted in a lower complexity score for the procedure compared to that for controls.^1^ Four studies with repeated measuring of total liver volume during hypocaloric diet treatment lasting between 4 and 12 weeks prior to MBS or anti‐reflux surgery, demonstrated maximal reduction of liver volume after 2,^33^, ^56^ 3,^57^ and 4 weeks.^47^ Thus, even if TBWL during hypocaloric diet treatment seems to continue decreasing after 4 weeks treatment, this might not be the case for changes in liver volume.

In addition to the reduction in liver volume, intrahepatic fat content was demonstrated to gradually decrease during 4 weeks of preoperative LED treatment.^33^ MASLD is linked to insulin resistance^58^ and increased fasting glucose concentrations.^59^ Moreover, hyperglycemia has been associated with adverse outcomes in surgical patients with and without diabetes in some studies,^3^, ^60^ but not in others.^4^ According to the American Society for Metabolic and Bariatric Surgery guidelines, preoperative screening of HbA1c and fasting glucose for diabetes and prediabetes is recommended, and with perioperative management of hyperglycemia.^4^ Consequently, the observed reductions in glucose concentrations and insulin resistance after hypocaloric diets as reported in this systematic review imply an improved glucometabolic condition preoperatively.

One limitation of this systematic review was the inability to conduct a meta‐analysis, and hence, our results should be interpreted carefully. However, the synthesis without meta‐analysis for some of the outcome measures provided information on the magnitude and ranges of effects. We were unable to perform any synthesis at all regarding side effects, because of large methodological variations. Another limitation was the low number of randomized controlled studies included in this review. The majority of studies were within‐group control that might introduce bias such as ignoring changes due to natural variations over time. Nonetheless, the inclusion of several study designs was also a strength, reducing the risk of missing relevant studies. In clinical practice, preoperative hypocaloric diets are often prescribed without monitoring of adherence, and here, we therefore reported the prescribed energy content of the hypocaloric diets and not the consumed diet. Lack of reporting of adherence to the prescribed diet could be one reason for the large variation in TBWL between studies, pinpointing the need for randomized controlled studies with concomitant measuring of adherence using validated dietary assessment methods. However, reductions in liver volume and reduced insulin resistance were more consistent among the studies we reviewed. Moreover, we deem the included study patients to be representative of the population accepted for MBS, at least for patients with a BMI below 50 kg/m^2^. The rigorous methodological process performed by the review team is definitely a strength of this systematic review. Finally, the study protocol was registered in the PROSPERO database for systematic reviews, which aims to reduce the opportunity for reporting bias and enables comparison of the completed review with the protocol.

The conclusion of this systematic review is that preoperative LED containing 800–1200 kcal/day probably result in similar TBWL as VLED containing 450‐ < 800 kcal/day, but that longer treatment periods seem to induce higher TBWL in patients undergoing MBS. In addition, a LED for 2–4 weeks might enhance patients' health status by reduction of liver volume and reduced insulin resistance preoperatively. Avoidance of excessive caloric restriction and treatment duration might prevent loss of lean body mass and patient attrition. Therefore, we suggest that both healthcare providers and manufacturers should consider whether to continue to prescribe and produce VLED or whether LED are sufficient. In this systematic review, we were able to answer the review question regarding the effect of different hypocaloric diet regimens on TBWL but not regarding the reduction in liver volume, glucose and insulin concentrations, or side effects. Our results are based on limited information, and we urgently call for randomized controlled trials with a concomitant focus on side effects and adherence.

## CONFLICT OF INTEREST STATEMENT

The authors reported no conflict of interest related to the study.

## Supporting information


**Table S1.** Search terms and results of literature search in PubMed.
**Table S2**. Study characteristics.
**Table S3**. Risk of bias questions for the controlled intervention (between‐group control) studies.
**Table S4**. Risk of bias questions for the before and after (within‐group control) studies.
**Table S5**. Study characteristics related to the review question for 32 studies (33 articles) included in the systematic review.
**Figure S1**. Flow diagram of study selection.
**Table S6.** Median sample size, BMI and age within treatment categories and for all studies.
