# Supplementary material for: Low‐energy diets before metabolic bariatric surgery: A systematic review of the effect on total body weight, liver volume, glycemia and side effects
Source: Obes Rev. 2024 Dec 3;26(4):e13876. doi: 10.1111/obr.13876 (PMC11884969; doi:10.1111/obr.13876)
Supplement: Supplementary file 1 — Table S1. Search terms and results of literature search in PubMed. Table S2. Study characteristics. Table S3. Risk of bias questions for the controlled intervention (between‐group control) studies. Table S4. Risk of bias questions for the before and after (within‐group control) studies. Table S5. Study characteristics related to the review question for 32 studies (33 articles) included in the systematic review. Figure S1. Flow diagram of study selection. Table S6. Median sample size, BMI and age within treatment categories and for all studies. [file OBR-26-e13876-s001.pdf]

SUPPORTING INFORMATION

Low-energy diets before metabolic bariatric surgery: a systematic review of the effect on total body weight, liver volume, glycemia and side effects

Authors:

Inger Nilsen [1,2,3], Agneta Andersson [1], Anna Laurenius [4,5], Johanna Österberg [6,7]

[1]Department of Food Studies, Nutrition and Dietetics, Uppsala University, Uppsala, Sweden

[2]Center for Clinical Research Dalarna, Falun, Sweden

[3]Department of Dietetics and Speech Therapy, Mora Hospital, Mora, Sweden

[4]Department of Surgery, Institute of Clinical Science, Sahlgrenska Academy, University of Gothenburg, Gothenburg, Sweden

[5]Department of Gastroenterology and Hepatology, Unit of Clinical Nutrition and the Regional Obesity Center, Sahlgrenska University Hospital, Gothenburg Sweden

[6]Department of Surgery, Mora Hospital, Mora, Sweden

[7]Department of Clinical Science and Education, Södersjukhuset, Karolinska Institute, Stockholm, Sweden

Corresponding author: Inger Nilsen, Department of Food Studies, Nutrition and Dietetics, Uppsala University, BMC, Husargatan 3, Box 560, Uppsala, Sweden.

E-mail address: inger.nilsen@ikv.uu.se

Table S1. Search terms and results of literature search in PubMed.

Pubmed 2021-06-22			
	Search no	Search terms	Results
	#1	"bariatrics"[MeSH Terms] OR "obesity/surgery"[MeSH Terms] OR ”Weight Loss/Surgery”[MeSH Terms] OR ”Gastroenterostomy”[MeSH Terms] OR ”Gastrectomy”[MeSH Terms] OR ”Biliopancreatic Diversion”[MeSH Terms] OR ”Anastomosis, Roux-en-Y”[MeSH Terms]	69,775
	#2	"bariatric*"[Title/Abstract] OR "metabolic surg*"[Title/Abstract] OR "obesity surg*"[Title/Abstract] OR "weight loss surg*"[Title/Abstract] OR "gastric surg*"[Title/Abstract] OR "gastroenterestom*"[Title/Abstract] OR "gastroileal bypass"[Title/Abstract] OR "stomach stapling"[Title/Abstract] OR "gastric bypass"[Title/Abstract] OR "roux-en-y"[Title/Abstract] OR "rygb*"[Title/Abstract] OR "gastrectom*"[Title/Abstract] OR "gastric sleeve"[Title/Abstract] OR "ileal interposition"[Title/Abstract] OR "jejunoileal bypass"[Title/Abstract] OR "lap-band"[Title/Abstract] OR "gastric band"[Title/Abstract] OR "gastroplast*"[Title/Abstract] OR "biliopancreatic diversion*"[Title/Abstract] OR "biliopancreatic bypass"[Title/Abstract] OR "duodenojejunal bypass"[Title/Abstract] OR "duodenal jejunal bypass"[Title/Abstract] OR "duodenal switch"[Title/Abstract] OR "bpd ds*"[Title/Abstract] OR "sadi s*"[Title/Abstract] OR "duodenal-ileal bypass"[Title/Abstract] OR "duodeno-ileal bypass"[Title/Abstract]	65,229
	#3	#1 OR #2	89,877
	#4	"nutrition therapy"[MeSH Terms]	105,378
	#5	"diet therap*"[Title/Abstract] OR "nutrition therap*"[Title/Abstract] OR "diet reduc*"[Title/Abstract] OR "Ketogenic"[Title/Abstract] OR "mediterranean diet*"[Title/Abstract] OR "diet intervention*"[Title/Abstract] OR "low energy diet*"[Title/Abstract] OR "low calorie diet*"[Title/Abstract] OR "vled*"[Title/Abstract] OR "vlcd*"[Title/Abstract] OR "LCD"[Title/Abstract] OR "energy restriction*"[Title/Abstract] OR "energy reduc*"[Title/Abstract] OR "caloric restriction*"[Title/Abstract] OR "caloric reduc*"[Title/Abstract] OR "calorie restriction*"[Title/Abstract] OR "calorie reduc*"[Title/Abstract] OR "hypocalori*"[Title/Abstract] OR "weight loss diet*"[Title/Abstract] OR "weight reducing diet*"[Title/Abstract] OR "liquid diet*"[Title/Abstract] OR "meal replacement*"[Title/Abstract] OR "diet replacement*"[Title/Abstract]	38,582
	#6	#4 OR #5	128,291
	#7	"preoperative care"[MeSH Terms] OR "preoperative period"[MeSH Terms] OR "perioperative medicine"[MeSH Terms]	79,559
	#8	"Preoperative"[Title/Abstract] OR "Pre-operative"[Title/Abstract] OR "prehabilitati*"[Title/Abstract] OR "before surg*"[Title/Abstract] OR "presurg*"[Title/Abstract] OR "perioperative"[Title/Abstract] OR "peri-operative"[Title/Abstract]	419,967
	#9	#7 OR #8	455,248
	#10	"preoperative care"[MeSH Terms] OR "preoperative period"[MeSH Terms]	79,494
	#11	"Preoperative"[Title/Abstract] OR "Pre-operative"[Title/Abstract] OR "prehabilitati*"[Title/Abstract] OR "before surg*"[Title/Abstract] OR "presurg*"[Title/Abstract]	338,074
	#12	#10 OR #11	375,304
	#13	#3 AND #6 AND #9	420
	#14	#3 AND #6 AND #12	356

Table S2. Study characteristics.

Author	Publication year	Country	Study design	Intervention	Control	Outcomes	Groups included in systematic review
Albanese et al.	2019	Italy	Non-randomized control trial	700 kcal/day, 21 days<br>Very low-ketogenic and high-protein diet, breakfast and dinner replaced by whey protein powder enriched with amino acids and lunch including animal or vegetable protein and 200g vegetables outlined in diet scheme. Trace elements supplement. <20-30 grams carbohydrates/day	800 kcal/day, 21 days<br>Food- based diet, 5 meals, low-fat, diet outlined in diet scheme	Operative time, length of stay, drainage output, hemoglobin levels after surgery	Intervention and control group included in different treatment categories
Aukan et al.	2022	Norway	Non-randomized control trial	750 kcal/day, 14 days<br>Lighter Life diet replacement product, soups, shakes, pasta dishes, bars and porridge, 100 g of low-starch vegetables , 2.5 L water/day, non-caloric beverages allowed, max 500 mL low-energy drinks (<3 kcal/100 mL), 4 sugar-/calorie free chewing gum, artificial sweeteners, or mints/day	Non-surgical group treated with 10 weeks of the same very low-energy diet	Hedonic hunger and food reward	Intervention group included<br><br>Control group excluded according to eligibility criteria
Bakker et al.	2019	The Netherlands	Randomized control trial	Normal diet + omega-3 fatty acids	800 kcal/day, 14 days<br>Modifast diet replacement product, 1 fruit, 1 port clear broth, cooked vegetables (not legumes, corn, beetroot, avocado), raw vegetables (cucumber tomatoes, celery), water, tea, coffee (no milk or sugar), sugar free soda	Volume of the left liver lobe<br>Patient evaluation of LED, surgeons access to liver, muscle mass and visceral fat	Control group included<br><br>Intervention group excluded according to eligibility criteria
Baldry et al.	2017	UK	Randomized control trial	800 kcal/day, 14 days<br>Derby Teaching Hospital NHS Foundation Trust standard prebariatric surgery food-based diet	800 kcal/day, 14 days<br>Lighter Life diet replacement product	Liver histology<br>Total body weight, circulation inflammatory markers, peri-operative visual assessment of liver/perceived difficulty of surgery<br>Evaluation of diets	Intervention and control group included in the same treatment category

Author	Publication year	Country	Study design	Intervention	Control	Outcomes	Groups included in systematic review
Berggren et al.	2017	Sweden	Single-arm study	858 kcal/day, 25 days<br>Modifast diet replacement product	No	Glycemia, insulin, incretin response	Included as two groups in the same treatment category, normo-glycemic and Type 2 diabetes
Bennasar Remolar et al.	2016	Spain	Single-arm study	603 kcal/day, 28 days<br>Optifast diet replacement product, water, defatted stock and infusions	No	Total body weight and nutritional parameters: hemoglobin, lymphocytes, albumin	Intervention group included
Berk et al.	2019	Norway, The Netherlands	Non-randomized control trial	900 kcal/day, 49 days<br>Crisp bread and low-fat products for breakfast, lunch and supper. Dinner: fish, poultry, lean meat and potatoes, rice or pasta, multivitamin and mineral supplement	Non-surgical lifestyle group	Plasma Lipoprotein A and fatty acid levels	Intervention group included<br><br>Control group excluded according to eligibility criteria
Boshier et al.	2018	UK	Single-arm study	900 kcal/day, 21 days<br>Low-carbohydrate diet	No	Exhaled ketone concentrations and correlation to changes in body weight and nutritional markers	Intervention group included
Campos et al.	2010	USA	Non-randomized control trail	800 kcal/day, 14 days<br>Optifast HP diet replacement product, specific feeding schedule, non-caloric soft drinks and water	Low-energy diet after RYGB surgery	Total body glucose disposal, gut and pancreatic hormone secretion and body composition	Intervention group included<br><br>Control group excluded according to eligibility criteria
Chakravartty et al.	2019	UK	Randomized control trial	800 kcal/day, 28 days<br>Cambridge Milk diet: 3 pints (1,7 L) of semi skimmed milk, multivitamin and mineral supplement, ≥2 L energy free liquid (water, diet fizzy drinks, mineral water, black tea/coffee, or squash with no added sugar)	Continued with normal diet	Collagen gene expression and overall wound healing	Intervention and control group included
Cleveland et al.	2016	USA	Single-arm study	1,000 kcal/day, 14 days<br>150 kcal protein shake morning and afternoon and 400 kcal evening meal, handouts with instructions	No	Visceral adiposity	Intervention group included

Author	Publication year	Country	Study design	Intervention	Control	Outcomes	Groups included in systematic review
Davenport et al.	2019	Australia	Randomized control trial	636 kcal/day, 14 days Formulite (high protein) diet replacement product, 25 grams carbohydrates/day	657 kcal/day, 14 days Optifast diet replacement product	Compliance (urinary ketones) Total body weight, patient satisfaction, patient-reported outcomes, gastrointestinal side effects and surgical conditions	Intervention and control group included in the same treatment category
Edholm et al.	2011	Sweden	Non-randomized control trial	960 kcal/day, 28 days Modifast diet replacement product and water ad-libitum	Continued with normal diet	Liver volume and intrahepatic fat Facilitation of laparoscopic GBP	Intervention group included,<br><br>Control group excluded according to eligibility criteria
Edholm et al.	2015	Sweden	Single-arm study	800-1,100 kcal/day, 28 days Modifast diet replacement product	No	Liver volume, body composition and intrahepatic fat Patient-related outcomes: hunger, well-being, quality of life	Intervention group included
Ekici et al.	2019	Turkey	Non-randomized control trial	1,000 kcal/day, 28 days Prescription of a limiting high protein diet, verbal information	Patients that denied hypocaloric diet	Peri- and postoperative outcomes Liver size, complications and total body weight	Intervention and control group included
Erdem et al.	2022	Turkey	Non-randomized control trial	650 kcal/day, 15 days Societa Dietetica Medica Low-calorie ketogenic diet replacement product, 2-3L water, nutritional supplements, 44 grams carbohydrates/day	Mediterranean diet 1,630 kcal/day, 15 days	BMI Liver size, anthropometric parameters, metabolic outcomes	Intervention group included<br><br>Control group excluded according to eligibility criteria
Fris et al.	2006	New Zealand	Single-arm study	456 kcal/day, 14 days Optifast diet replacement product	No	Liver size Factors to predict reduction of liver size	Intervention group included
Gils Contreras et al.	2018	Spain	Randomized control trial	800 kcal/day, 21 days Optifast diet replacement product, broth and liquids with no calories	1,200 kcal/day, 21 days Food-based diet, 800 kcal from food and Optifast diet replacement product, 21 different daily menus	Liver volume Total body weight and composition, blood pressure, biochemical parameters, compliance, diet tolerance, surgery complications and length of stay	Intervention and control group included in the same treatment category
Gonzales-Perez et al.	2013	Mexico	Single-arm study	800 kcal/day, 42 days Food-based diet plan, freely vegetables with low energy density, 1.5-2 L of water per day 40 grams carbohydrates/day	No	Total body weight and liver size	Intervention group included

Author	Publication year	Country	Study design	Intervention	Control	Outcomes	Groups included in systematic review
Kullberg et al. (Same study as Edholm et al. 2011)	2011	Sweden	Single-arm study	960 kcal/day, 28 days<br>Modifast diet replacement product and water ad-libitum	No	Lipid dynamics<br>Total amounts of body fat and liver fat	Intervention group included
Lange et al.	2022	Germany	Randomized control trial	913 kcal/day, 14 days<br>BCM Diät replacement product, 200 g/day low-starch vegetables, 2 L/day calorie-free beverages	841 kcal/day, 14 days<br>Optifast diet replacement product, 200 g/day low-starch vegetables, 2 L/day calorie-free beverages	Liver volume and liver fat content<br>Total body weight, excess weight and waist circumference<br>Visceral and subcutaneous adipose tissue mass, laboratory parameters, adherence to and acceptance of diet and side effects	Intervention and control group included in the same treatment category
Nielsen et al.	2015	Denmark	Single-arm study	1,030 kcal/day, 49 days<br>Cambridge Weight Plan consisting of 4 powder-based meals and 1L skimmed milk, 295 g vegetables, 100 g low-fat yoghurt/day and RDA for vitamins and minerals	No	Total body weight<br>Anthropometric and biochemical measures, blood pressure, heart rate and side effects	Intervention group included
Norén et al.	2014	Sweden	Single-arm study	680 kcal/day, 28 days<br>SLANKA diet replacement product	No	Metabolic variables: glucose, HbA1c, cholesterol, triglycerides, blood pressure, total body weight	Intervention group included
Pournaras et al.	2016	UK	Randomized control trial	800 kcal/day, 14 days<br>Optifast diet replacement product	Continued with normal diet	Whole body glucose disposal, substrate utilization and postprandial glucose and insulin response	Intervention and control group included
Pösö et al.	2013	Sweden	Single-arm study	600 kcal/day, 21 days<br>Allevo, Nutrilett or Naturdiet diet replacement product	No	Preoperative transthoracic echocardiography before and after intravascular volume challenge	Intervention group included
Schiavo et al.	2015	Italy	Single-arm study	1,200 kcal/day, 56 days<br>Food-based Mediterranean diet	No	Total body weight, visceral fat, liver size, fat mass and fat free mass	Intervention group included
Schiavo et al.	2018	Italy	Single-arm study	1,200 kcal/day, 28 days<br>Food-based, micronutrient-enriched ketogenic diet plans, <20grams carbohydrates/day	No	Total body weight, left hepatic lobe volume and micronutrient status	Intervention group included

Author	Publication year	Country	Study design	Intervention	Control	Outcomes	Groups included in systematic review
Schiavo et al.	2022	Italy	Randomized control trial	1,150-1,250 kcal/day, 28 days, Food–based low-calorie ketogenic diet, meal plan 1 days 1-14 and meal plan 2 days 15-28 and assigned individual foods. Supplement Ketocompleat.<br><20g carbohydrate/day	Continued with normal diet	Apnea-hypopnea index<br>Total body weight, hypertension, dyslipidemia, insulin resistance, CRP	Intervention and control group included
Sen et al.	2021	Turkey	Single-arm study	1,000 kcal/day, 14 days	No	Operative time, LOS, early postoperative complications and postoperative anthropometry	Intervention group included
Sivakumar et al.	2020	Australia	Single-arm study	600 kcal/day, 14 days<br>Optifast diet replacement product, low-starch vegetables, 1 teaspoon olive oil	No	Total body weight, excess body weight, BMI, body composition (fat mass, lean body mass and bone mineral content)	Intervention group included
Van Nieuwenhove et al.	2011	The Netherlands, Sweden, Lithuania, Spain, Belgium	Randomized control trial	800 kcal/day, 14 days<br>Optifast diet replacement product	Continued with normal diet	Operating time, surgeon´s perceived difficulty of the operation, liver lacerations, intraoperative bleeding and complications, 30-day total body weight and morbidity	Intervention and control group included
Wolf et al.	2019	USA	Non-randomized control trial	1,200 kcal/day, 14 days<br>Low-carbohydrate (amount not reported), high protein shakes for breakfast and lunch and dinner consisting of 3-6 oz. protein, 1 cup vegetable and 2 snacks (a designated fruit, 6-oz light Greek yoghurt or protein shake)	Continued with normal diet	Liver histology	Intervention and control group included
Yolsuriyanwong et al.	2019	USA	Single-arm study	800 kcal/day, 14 days<br>Atkins diet replacement product or similar, detailed diet instructions and daily vitamin, minerals and trace elements. One serving size per day of sugar-free fluid (sugar-free popsicle, sugar-free gelatin or a cup of broth containing <20 kcal). 25 grams carbohydrates/day	No	Total body weight<br>Compliance and acceptability	Patient group with BMI≥50 kg/m^2 included<br><br>Patient group with lower BMI<50 kg/m^2 excluded according to eligibility criteria

Table S3. Risk of bias questions for the controlled intervention (between-group control) studies.

Item		Yes	No	Not reported/ Cannot determine
1	Was the study described as randomized, a randomized trial, a randomized clinical trial, or an RCT?			
2	Was the method of randomization adequate (i.e., use of randomly generated assignment)?			
3	Was the treatment allocation concealed (so that assignments could not be predicted)?			
4	Were study participants and providers blinded to treatment group assignment?			
5	Were the people assessing the outcomes blinded to the participants' group assignments?			
6	Were the groups similar at baseline on important characteristics that could affect outcomes (e.g., demographics, risk factors, co-morbid conditions)?			
7	Was the overall drop-out rate from the study at endpoint 20% or lower of the number allocated to treatment?			
8	Was the differential dropout rate (between treatment groups) at endpoint 15 percentage points or lower?			
9	Was there high adherence to the intervention protocols for each treatment group?			
10	Were other interventions avoided or similar in the groups (e.g., similar background treatments)?			
11	Were outcomes assessed using valid and reliable measures, implemented consistently across all study participants?			
12	Did the authors report that the sample size was sufficiently large to be able to detect a difference in the main outcome between groups with at least 80% power? (regarding the outcomes in this systematic review)			
13	Were outcomes reported or subgroups analyzed pre-specified (i.e., identified before analyses were conducted)?			
14	Were all randomized participants analyzed in the group to which they were originally assigned, i.e., did they use an intention-to-treat analysis?			
15	Was there no conflict of interest related to the study?			

Table S4. Risk of bias questions for the before and after (within-group control) studies.

Item		Yes	No	Not reported/ Cannot determine
1	Was the study question or objective clearly stated?			
2	Were eligibility/selection criteria for the study population pre-specified and clearly described?			
3	Were the participants in the study representative of those who would be eligible for the test/service/intervention in the general or clinical population of interest?			
4	Were all eligible participants that met the pre-specified entry criteria enrolled?			
5	Was the sample size sufficiently large to provide confidence in the findings?			
6	Was the test/service/intervention clearly described and delivered consistently across the study population?			
7	Were the outcome measures pre-specified, clearly defined, valid, reliable, and assessed consistently across all study participants?			
8	Were the people assessing the outcomes blinded to the participants' exposures/interventions?			
9	Was the loss to follow-up after baseline 20% or less?			
10	Did the statistical methods examine changes in outcome measures from before to after the intervention? Were statistical tests done that provided p values for the pre-to-post changes?			
11	Were outcome measures of interest taken multiple times before the intervention and multiple times after the intervention (i.e., did they use an interrupted time-series design)?			
12	Was there no conflict of interest related to the study?			

Table S5. Study characteristics related to the review question for 32 studies (33 articles) included in the systematic review.

Author, publication year	Number of patients included, N	Dropouts after inclusion, N	Age, years	Gender, M/F	No diabetes/ Type 2 diabetes	BMI baseline, kg/m^2	Outcomes for systematic review			
							TBWL	Reduction in liver volume	Reduction in fasting glucose and/or insulin concentrations	Side effects
Chakravartty et al., 2019, interv./control	15/13	5/3	43.5†/38.5†	1/9 / 0/10	10/0 / 10/0	53.4†/52.8†	√	√		
Ekici et al., 2019, interv./control	128	27	37.4 ± 9.2/37.5 ± 9.6	17/32 / 19/33	28/21 / 31/21	45.1 ± 4.4/44.9 ± 4.1	√			
Pournaras et al., 2016, interv./control	7/8	0/0	49.3 ± 23.0/44.2 ± 6.4	4/3 / 3/5	0/7 / 0/8	40.1 ± 3.8/42.0 ± 4.5	√		√	
Schiavo et al., 2022, interv./control	41/41	7/5	42 ± 13.7 (interv. + control)	22/12 / 22/14	NR	50.1 ± 5.9/47.6 ± 5.9	√		√	
Van Niewenhove et al., 2011, interv./control	149/145	12/7	39.7 ± 9.5/40.3 ± 9.7	40/97 / 41/95	117/19 / 118/19	43.4 ± 10.0/43.3	√			
Wolf et al., 2019, interv./control	20/20	0/0	45.5/37.9	1/19 / 4/16	NR	48.3/48.0	√			
Albanese et al., 2019, Group 1	106	0	43.5 ± 11.8	27/79	77/29	43.6 ± 6.9	√			
Bakker et al., 2019	31	5	44 (18) †	0/26	19/7	41 (6)†	√	√		√
Baldry et al., 2017, Group 1	30	4	47.0†	4/22	18/8	51.10†	√			√
Baldry et al., 2017, Group 2	30	2	42.0†	6/22	20/8	50.10†	√			√
Berggren et al., 2017	19	NR	43.0 ± 7.4	0/19	9/10	40.2 ± 3.6 (no diabetes)/ 39.4 ± 3.5 (type 2 diabetes)	√		√	
Boshier et al., 2018	40	0	50.7 ± 11.4	9/31	16/24	45.1 ± 7.5	√			
Campos et al., 2010	10	NR	40.2 ± 13.4	4/6	10/0	48.3 ± 6.6	√		√	
Cleveland et al., 2016	40	NR	37.5	3/37	NR	42.70	√			
Edholm et al., 2011/ Kullberg et al., 2011	15	0	34.3 ± 7.5	0/15	NR	42.9 ± 3.0	√	√	√	
Edholm et al., 2015	10	0	43 ± 8.9	0/10	NR	41.7 ± 2.6	√	√	√	√
Gils Contreras et al., 2018, Group 1	43	0	45.2 ± 10.5	14/29	32/11	47.3 ± 5.3	√	√	√	√
Gils Contreras et al., 2018, Group 2	43	2	45.5 ± 9.7	7/34	37/4	47.2 ± 5.0	√	√	√	√

Caption: Data are mean ± SD or *95%CI or †median (IQR). N = number of patients; M = male; F = female; BMI = body mass index; TBWL = total body weight loss; interv. = intervention; NR = not reported; SD = standard deviation; CI = confidence interval; IQR = interquartile range.

Table S5 continued. Study characteristics related to the review question for 32 studies (33 articles) included in the systematic review.

Author, publication year	Number of patients included, N	Dropouts after inclusion, N	Age, years	Gender, M/F	No diabetes/ Type 2 diabetes	BMI baseline, kg/m²	Outcomes for systematic review			
							TBWL	Reduction in liver volume	Reduction in fasting glucose and/or insulin concentrations	Side effects
Lange et al., 2022, Group 1	45	12	47.2 ± 11.5	11/22	17/17	47.5 ± 5.5	√	√		√
Lange et al., 2022, Group 2	45	9	46.3 ± 8.8	11/25	19/17	47.5 ± 5.0	√	√		√
Schiavo et al., 2018	27	0	41.0 ± 16.7	10/17	21/6	44.5 ± 10.5(M)/46.9 ± 11.7(F)	√	√	√	√
Sen et al., 2021	305	0	37.8 ± 11.9	143/162	272/33	41.7 ± 6.9	√			
Yolsuriyanwong et al., 2019	34	NR	45.3 ± 12.9	7/27	NR	57.2 ± 6.0	√			√
Albanese et al., 2019, Group 2	72	0	43.4 ± 12.1	12/60	58/14	46.0 ± 6.3	√			
Aukan et al., 2022	37	17	NR	NR	NR	40.8 (38.7-42.8)*	√			
Bennasar Remolar et al., 2016	50	8	43.9	13/29	NR		√			
Davenport et al., 2019, Group 1	35	0	41.2 ± 12.9	4/31	34/1	42.2 ± 19.6	√			√
Davenport et al., 2019, Group 2	34	0	40.7 ± 13.6	2/32	31/3	43.0 ± 8.1	√			√
Erdem et al., 2022	25	10	42.9 ± 12.6	4/11	NR	47.80†	√		√	
Fris et al., 2004	50	10	41.5 ± 10.4	5/35	36/4	45.3 ± 6.9	√			
Norén et al., 2014	25	0	48.8	2/23	18/7	39.80	√		√	
Pösö et al., 2013	34	0	42.8 ± 8.8	11/23	22/12	45.90	√			
Sivakumar et al., 2020	60	16	43.5 (14)	11/33	35/9	43.4 ± 5.6	√			
Berk et al., 2019	98	16	41 (13)†	28/54	58/24	45.6 ± 5.3	√		√	
Gonzales-Perez et al., 2013	20	0	34.5† ± 11.5	3/17	NR	46.0† ± 5.3	√	√		√
Nielsen et al., 2015	30	2	38.8 ± 10.4	8/22	28/0	46.0 ± 4.4	√		√	√
Schiavo et al., 2015	37	NR	46 ± 7.1	37/0	NR	45.2 ± 4.9	√		√	√

Caption: Data are mean ± SD or *95%CI or †median (IQR). N = number of patients; M = male; F = female; BMI = body mass index; TBWL = total body weight loss; interv. = intervention; NR = not reported; SD = standard deviation; CI = confidence interval; IQR = interquartile range.

Figure S1. Flow diagram of study selection.

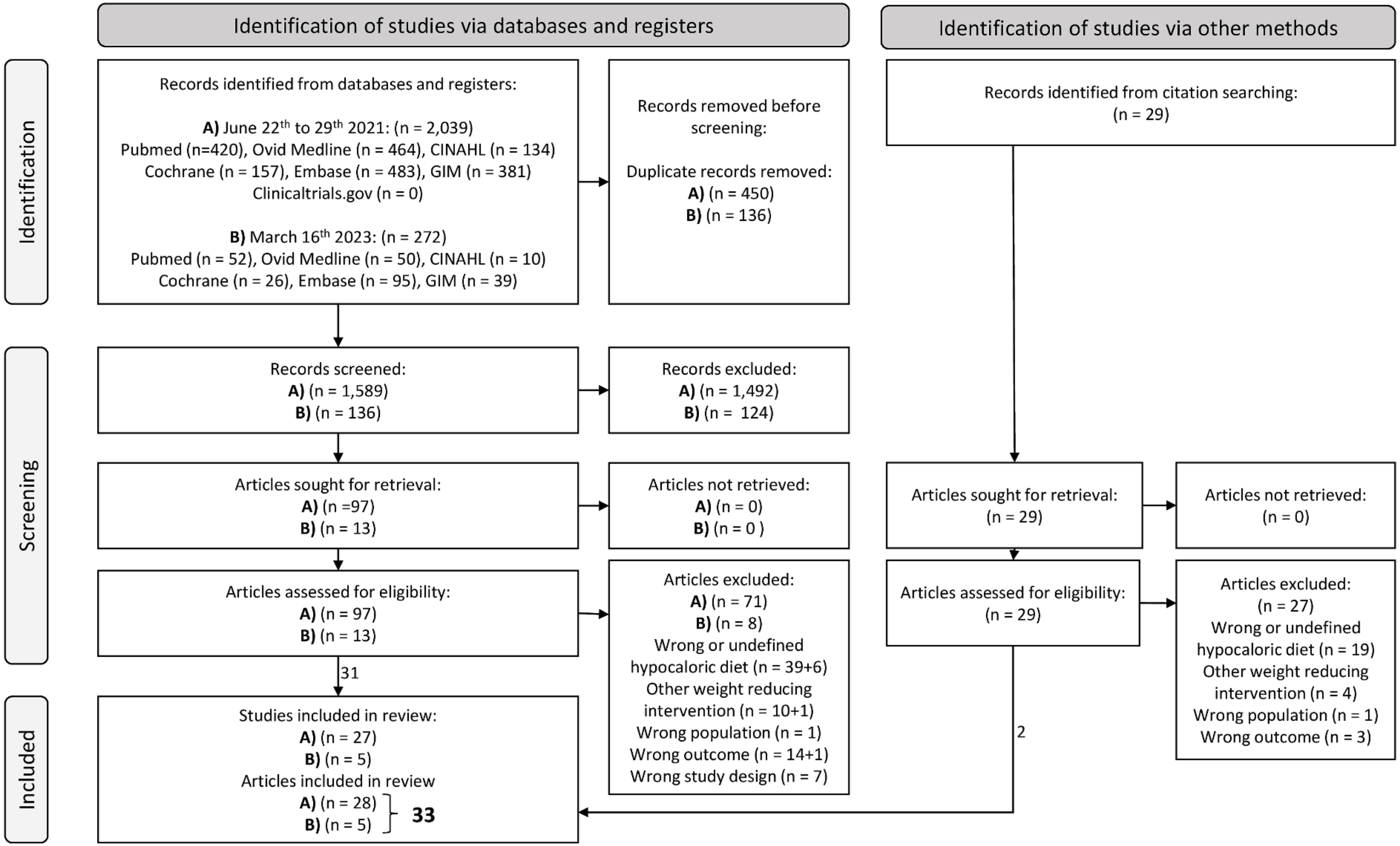

Table S6. Median sample size, BMI and age within treatment categories and for all studies.

	LED short duration vs. normal diet	LED short duration	VLED short duration	LED long duration	Other	All studies
Number of studies	6	13	9	4	(1)	32 (33)
Sample size, N<br>Median<br>(25-75-percentiles)<br>Min-Max	 27<br>(13-45)<br>7-137	 28<br>(13-40)<br>9-305	 35<br>(27-42)<br>15-72	 34<br>(28-48)<br>20-82		 34<br>(19-41)<br>7-305
BMI, kg/m^2<br>Median<br>(25-75-percentiles)<br>Min-Max	 46.7<br>(43.8-49.7)<br>40.1-53.4	 45.1<br>(42.2-47.5)<br>39.4-57.2	 43.4<br>(42.2-45.9)<br>39.8-47.8	 45.8<br>(45.5-46.0)<br>45.2-46.0		 45.3<br>(42.8-47.4)<br>39.4-57.2
Age, years<br>Median<br>(25-75-percentiles)<br>Min-Max	 42.8<br>(40.3-45.0)<br>37.4-49.3	 43.5<br>(41.0-45.5)<br>34.2-50.7	 42.9<br>(41.5-43.5)<br>40.7-48.8	 39.9<br>(37.7-42.3)<br>34.5-46.0		 43.0<br>(40.9-45.3)<br>34.3-50.7

LED short duration vs. normal diet is 800-1,200 kcal/d, 2-4 weeks vs. normal diet; LED short duration is 800-1,200 kcal/d, 2-4 weeks; VLED short duration is 450-<800 kcal/d, 2-4 weeks; LED long duration is 800-1,200 kcal/d, >4 weeks. LED = low energy diet; VLED = very low energy diet; BMI = body mass index.
